# 6-(2-Aminoethyl)-6*H*-indolo[2,3-*b*]quinoxalines as Promising Compounds Capable of Binding to FLT3 (D835V) Kinase

**DOI:** 10.3390/biom16081173

**Published:** 2026-08-12

**Authors:** Igor A. Schepetkin, Alexander V. Uvarov, Egor A. Evriinov, Andrei I. Khlebnikov

**Affiliations:** 1 Department of Microbiology and Cell Biology, Montana State University, Bozeman, MT 59717, USA; 2 Kizhner Research Center, Tomsk Polytechnic University, Tomsk 634050, Russia

**Keywords:** FMS-like tyrosine kinase 3 (FLT3), 6-(2-aminoethyl)-6*H*-indolo[2,3-*b*]quinoxalines, anticancer, molecular docking, molecular dynamics, tyrosine kinase, kinase inhibitor

## Abstract

Indolo[2,3-*b*]quinoxalines, along with their *N*-substituted derivatives, exhibit pronounced anticancer activity, although the mechanisms of their biological action may vary. Herein, a panel of sixty-five 6-(2-aminoethyl)-6*H*-indolo[2,3-*b*]quinoxaline derivatives comprising eight series with distinct amine moieties connected to the tetracyclic indoloquinoxaline core via a dimethylene linker was evaluated as drug-like candidates for kinase binding and cytotoxic activity. The ADME (Absorption, Distribution, Metabolism, and Excretion) properties of the compounds included in this set were preliminarily determined using the SwissADME tool. Analysis revealed that the library of quinoxaline derivatives largely complies with the drug-likeness rule for kinase-targeted compounds. As part of the biological screening, the compounds were initially tested on two cell lines MonoMac-6 and THP-1 (both derived from patients with acute monocytic leukemia) using sunitinib, a known antitumor agent acting as a multi-target receptor tyrosine kinase inhibitor, as a reference compound. Compound **3g**, which demonstrated the highest activity in the cytotoxicity analysis (IC_50_ = 1.9 and 3.5 μM for the MonoMac-6 and THP-1 cell lines, respectively), was screened using the Eurofins DiscoverX scanEDGE panel, comprising 97 distinct kinases representing all known kinase families. Subsequently, the compound was tested using the Eurofins DiscoverX scanTK™ panel, covering 135 distinct receptor and non-receptor tyrosine kinases. Based on initial screening results, compound **3g** exhibits relatively high binding activity against fourteen tyrosine kinases, including TYK2, ZAP70, eight mutant forms of ABL1, two mutant forms of FLT3, and one mutant form of ALK, and demonstrates relatively high binding selectivity with respect to non-mutant tyrosine kinases (S-score: 0.024). Secondary screening of nine selected analogs of compound **3g** led to the identification of compound **3h**, which demonstrates relatively high binding affinity for FLT3 (D835V) (K_d_ = 0.41 μM). Molecular modeling suggested modes of binding interaction of the compounds **3h** and **3g** in the FLT3 (D835V) catalytic site. Our results demonstrate that 6-(2-aminoethyl)-6*H*-indolo[2,3-*b*]quinoxaline derivatives could be potential candidates for developing anticancer drugs.

## 1. Introduction

Quinoxaline derivatives are versatile nitrogen-containing heterocycles widely used for their medicinal properties, including antimicrobial, anti-inflammatory, and anticancer activities [1,2]. Several quinoxaline-based analogs are FDA-approved anticancer drugs, including tyrosine kinase inhibitors erdafitinib and tyrphostins (AG1295 and AG1296) [2]. Some quinoxaline derivatives have been described as cyclooxygenase-2 (COX-2) inhibitors and may be used as anti-inflammatory agents [3]. Due to their planar, aromatic ring system, quinoxaline derivatives can intercalate between DNA base pairs and, through this interaction, inhibit topoisomerase activity [4]. Indoloquinoxalines are a specialized subclass of quinoxaline derivatives featuring a fused tetracyclic system, typically merging an indole ring with a quinoxaline nucleus. Depending on the mode of fusion, these compounds are classified into two major chemical subclasses: indolo[2,3-*b*]quinoxalines (linearly fused, also known as 6*H*-indolo[2,3-*b*]quinoxalines) and indolo[1,2-*a*]quinoxalines (angularly fused). Both subclasses, along with their N-substituted derivatives, exhibit significant biological activity, with a particularly strong emphasis on anticancer effects [5,6,7,8,9,10,11].

**Figure 1:**
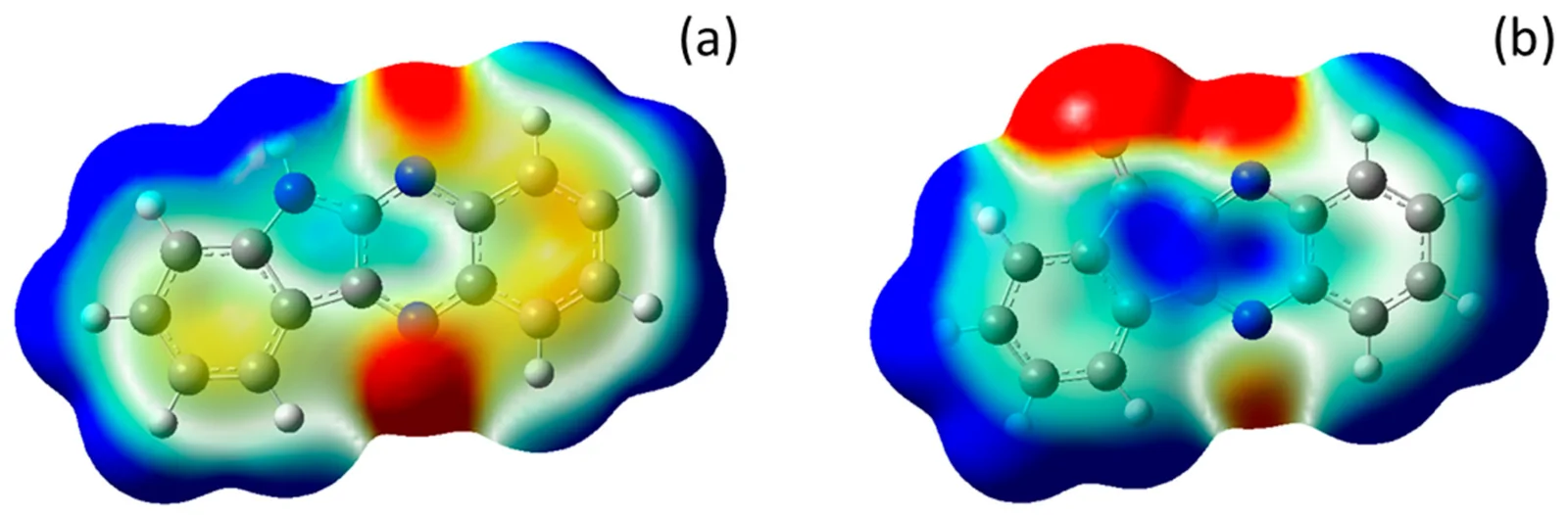
Molecular electrostatic potential maps generated for 6*H*-indolo[2,3-*b*]quinoxaline (**a**) and 11*H*-indeno[1,2-*b*]quinoxalin-11-one (**b**) scaffolds. The blue color corresponds to the electron-deficient regions, and the yellow to red colors represent the electron-rich regions. The electron density isosurface value is 0.0004 a.u. The data were obtained by the DFT method at the B3LYP/6-31+G(d) level.

The planar aromatic system of 6*H*-indolo[2,3-*b*]quinoxaline (Figure 1a) facilitates DNA intercalation and/or topoisomerase inhibition, which are considered the predominant mechanisms of their pharmacological action, including antiviral and cytotoxic [12,13], although other mechanisms of action cannot be ruled out. For example, certain 5-substituted 2-bromoindolo[2,3-*b*]quinoxalines (analogs of the antiviral compound B-220) exhibited anticancer activity (in vitro), as well as the ability to inhibit cyclin-dependent kinase 1 (CDK1) and Cdc25 phosphatase [14]. Indeed, planar fused heterocyclic scaffolds rank among the most successful “privileged structures” in medicinal chemistry employed for kinase inhibition, because these scaffolds can optimally occupy the ATP-binding cleft of a protein kinase [15,16,17]. An example of this is the 11*H*-indeno[1,2-*b*]quinoxalin-11-one scaffold, which also possesses a rigid, planar structure (Figure 1b), whose derivatives have demonstrated high binding affinity for kinases, including c-Jun N-terminal kinases 1-3 (JNK1-3), casein kinase 1 (CK1), phosphatidylinositol 3-kinase γ (PI3K-γ), and mitogen-activated protein kinase (MAPK)-interacting serine/threonine-protein kinase 2 (MKNK2) [18]. It should also be noted that powerful DNA-intercalating agents with a planar ring framework, ellipticine and ellipticinium, were predicted to act as inhibitors of FMS-like tyrosine kinase 3 (FLT3) [19].

FLT3 is a validated and highly active kinase target in the development of anticancer compounds, particularly for treating acute myeloid leukemia (AML) [20,21,22,23]. FLT3 mutations are detected in ~30% of newly diagnosed AML and 30–40% of acute promyelocytic leukemia [24,25]. They represent point mutations in the tyrosine kinase domain (TKD), most frequently at position D835, which stabilize the active kinase conformation [26,27]. These mutations lead to constitutive activation of the receptor and enhanced signaling via phosphoinositide 3-kinase/Akt kinase and STAT5 (signal transducer and activator of transcription 5) pathways in the absence of FLT3 ligand [28,29]. For example, the mutant form of FLT3 (ITD, D835V) is involved in the development of early blood cells [30]. The mutations, an internal tandem duplication (ITD) and a point mutation at residue 835 (D835V), cause the kinase to be constitutively active, meaning it is always “on” and promotes the survival and proliferation of cancer cells in AML. This dual mutation is associated with a poor prognosis [30,31,32].

In the present study, we evaluated the cytotoxic activity of a series of 6-(2-aminoethyl)-6*H*-indolo[2,3-*b*]quinoxalines and attempted to identify kinase targets for several selected compounds from this series. For the biological screening of the selected library of sixty-five 6-(2-aminoethyl)-6*H*-indolo[2,3-*b*]quinoxaline derivatives, we evaluated their cytotoxic effects in two cell lines, including THP-1 and MonoMac-6 cells, using sunitinib, a multi-targeted receptor tyrosine kinase (RTK) inhibitor, as a positive control. The most active compound **3g** (9-nitro-6-(2-(piperidin-1-yl)ethyl)-6*H*-indolo[2,3-*b*]quinoxaline), identified during a screen for cytotoxic activity, was subsequently tested against two kinase panels; the results indicate that the mutant kinase FLT3 (D835V) is a likely biological target for this compound. Furthermore, several additional analogs of compound **3g** were tested for binding affinity toward FLT3 (D835V). Importantly, the molecular modeling results regarding the binding modes of the two selected compounds to wild-type FLT3 and FLT3 (D835V) proteins are consistent with experimental data, confirming that 6-(2-aminoethyl)-6*H*-indolo[2,3-*b*]quinoxaline is a promising structural scaffold for the development of FLT3 inhibitors with antitumor activity.

## 2. Materials and Methods

### 2.1. Studied Compounds

Sixty-five derivatives of 6-(2-aminoethyl)-6*H*-indolo[2,3-*b*]quinoxalines were provided by Dr. Sergey A. Lyakhov from the A.V. Bogatsky Physico-Chemical Institute of the National Academy of Sciences of Ukraine (Odessa, Ukraine). Compounds **1a**, **1i**, **3a**, **4a**, **5a**, **6a**, **7a**, and **8a** were synthesized as described previously [9,33]. According to NMR spectroscopy and mass spectrometry data, the purity of the compounds was over 99%. The reference anticancer compound sunitinib was obtained from R&D Systems (cat. # 3768; Minneapolis, MN, USA). The compounds were dissolved in dimethyl sulfoxide (DMSO) at a concentration of 10 mM.

### 2.2. Biological Assays

#### 2.2.1. Cell Culture

Human THP-1 monocytic cells were obtained from InvivoGen (San Diego, CA, USA). These cells were cultured in RPMI 1640 medium (Mediatech Inc., Herndon, VA, USA) containing 10% (*v*/*v*) fetal bovine serum (FBS; cat. # F4135), 100 μg/mL streptomycin (cat. # SBR00068), and 100 U/mL penicillin (cat. # P3032); all from Sigma-Aldrich (St. Louis, MO, USA). Human MonoMac-6 monocyte/macrophage cells (Deutsche Sammlung von Mikroorganismen und Zellkulturen GmbH, Braunschweig, Germany) were cultured in RPMI 1640 medium containing 10% (*v*/*v*) FBS, 10 μg/mL bovine insulin, 100 μg/mL streptomycin, and 100 U/mL penicillin. Human monocytic THP-1 cells express a functional, wild-type form of FLT3 on their cell surface, with an apparent molecular mass of 150 kDa [34]. MonoMac-6 cells carry an activating point mutation at codon 592 in the juxtamembrane domain of FLT3 (FLT3-V592A) [35]. Both THP-1 and MonoMac-6 cell lines express the wild-type (normal) ABL1 kinase but generally do not express oncogenic, mutant forms of the ABL1 gene [36].

#### 2.2.2. Cytotoxicity Assay

Cytotoxicity was analyzed with a CellTiter-Glo Luminescent Cell Viability Assay Kit from Promega (cat. # G9241; Madison, WI, USA) according to the manufacturer’s protocol. THP-1 cells were treated with varying concentrations of the test compounds (up to 100 µM) and cultivated for 24 h. After treatment, the cells were allowed to equilibrate to room temperature for 30 min, the substrate was added, and the samples were analyzed with a Fluoroscan Ascent FL (Thermo Fisher Scientific, Waltham, MA, USA). The concentration of the compound causing 50% cytotoxicity relative to the control (1% DMSO) was calculated by plotting the percentage of inhibition against the logarithm of the inhibitor concentration (IC_50_).

#### 2.2.3. Kinase Profiling and K_d_ Determination

Kinase profiling of compound **3g** was performed by Eurofins Discovery (San Diego, CA, USA) at a concentration of 10 µM, using (1) scanEdge panel of 97 protein kinases and (2) scanTK™ panel of 135 tyrosine kinases, as described previously [37]. Selected compounds were also submitted for dissociation constant (K_d_) determination toward the target mutated tyrosine kinase FLT3 (D835V) using Eurofins Discovery. In brief, kinases were produced and displayed on T7 phages or expressed in HEK-293 cells. Binding reactions were performed at room temperature for 1 h, and the fraction of kinase not bound to the test compound was determined by capture with an immobilized affinity ligand and quantified by quantitative polymerase chain reaction. The primary screening at fixed concentrations of the compound was performed in duplicate. For dissociation constant K_d_ determination, a 12-point half-log dilution series (a maximum concentration of 33 μM) was used. The parameter K_d_ represents the molar concentration of the inhibitor at which, at equilibrium, 50% of the binding sites of the target kinase are occupied by the compound under investigation. Assays were performed in duplicate, and their average mean values are displayed.

### 2.3. Computer Modeling and Simulation

#### 2.3.1. Molecular Docking

The three-dimensional structures of compounds **3h** and **3g** were built using ChemOffice 2016 software (PerkinElmer Informatics, Inc., Waltham, MA, USA) and optimized with the MM2 force field [38]. The crystal structures of FLT3 kinase in DFG-out and DFG-in conformations were downloaded from the Protein Data Bank (PDB IDs: 4RT7 and 6JQR, respectively). The missing loop (residues 831–836) of 6JQR was added using the SWISS-MODEL web server (https://www.swissmodel.expasy.org, accessed on 6 April 2026) based on the template structure of KIT (PDB ID: 1PKG), which shares 56% identity with FLT3 [39]. Molecular docking was performed using Molegro Virtual Docker (MVD) version 6.0 (CLC bio, Qiagen, Aarhus, Denmark). To build D835V mutants, the enzyme structures were modified in MVD, and the amino acid residues lying within 8 Å from the mutated residue were optimized with the MolDock force field.

The FLT3 structures and ligand models were imported into the MVD workspaces. To define the search space, spherical areas with radii of 12 Å were placed on the centroid positions of the co-crystallized molecules quizartinib or gilteritinib. For each of the investigated ligands, 500 independent docking runs were executed with each enzyme structure. The “Internal HBond” and “sp^2^-sp^2^ Torsions” ligand evaluation options were enabled during the calculations. The MolDock scoring function [40] was applied to evaluate the obtained poses. The results were analyzed and visualized with the MVD built-in tools.

#### 2.3.2. Molecular Dynamics (MD) Simulation

MD simulation was performed on the top-scoring docked model using NAMD 3.1 software (University of Illinois, Urbana, IL, USA) [41] with the AMBER ff19SB force field [42]. The protein was protonated at physiological pH (7.4) using PROPKA 3.5.1 (University of Copenhagen, Copenhagen, Denmark) [43,44] for pK_a_ prediction and PDB2PQR 3.7.1 (Pacific Northwest National Laboratory, Richland, WA, USA) [45,46,47,48] for hydrogen placement under the AMBER naming convention, followed by cleanup with pdb4amber utility (University of California, San Francisco, CA, USA) [49] to remove crystallographic waters and ensure AMBER-compatible residue names. The system was subsequently immersed in an 89 × 74 × 96 Å^3^ box of OPC (Optimal Point Charge) water molecules [50] and neutralized with NaCl added to a physiological concentration of 150 mM. The ligand **3h** was parametrized with the GAFF2 force field [51] using AM1-BCC [52] partial charges. Following preparation, the system was energy-minimized for 5000 steps and subjected to NVT heating from 50 K to 310 K over 5 ns. An NPT relaxation phase with progressively released positional restraints was then carried out for 5 ns, followed by an unrestrained NPT production run of 100 ns.

#### 2.3.3. ADME Calculations and Regression Analysis

ADME parameters were computed using the SwissADME web server (https://swissadme.ch, accessed on 30 March 2026). Among the calculated descriptors, Silicos-IT Log P values were extracted for subsequent analysis. Linear correlation between these calculated lipophilicity estimates and the corresponding experimental activity data (expressed as −log IC_50_) was assessed using the built-in regression tools in Microsoft Excel.

#### 2.3.4. DFT Calculations

The structures of 6*H*-indolo[2,3-*b*]quinoxaline and 11*H*-indeno[1,2-*b*]quinoxaline-11-one were optimized in the gas phase using density functional theory (DFT) with the Gaussian 09 software package (Gaussian, Inc., Wallingford, CT, USA). The B3LYP functional and the 6-31+G(d) basis set were employed. The identification of true energy minima was confirmed by the absence of imaginary frequencies during vibrational analysis. The molecular electrostatic potential (ESP) was mapped onto an electron density isosurface (0.0004 a.u.) using GaussView 6 (Gaussian, Inc., Wallingford, CT, USA). The color scale in Figure 1 was adjusted to an ESP range from −0.025 a.u. (red) to +0.010 a.u. (blue).

#### 2.3.5. Disclosure of AI-Assisted Language Editing

During the preparation of this work, the authors used Claude Sonnet-4.6 to polish the English language and improve readability. After using this tool, the content was reviewed and edited as needed, and the authors take full responsibility for the final version.

## 3. Results and Discussion

### 3.1. General Characterization of Small Library of 6-(2-Aminoethyl)-6H-indolo[2,3-b]quinoxalines

In the present study, we utilized a small library of chemically related 6-(2-aminoethyl)-6*H*-indolo[2,3-*b*]quinoxaline derivatives comprising sixty-five drug-like molecules (Table 1). A number of these compounds, including **1a**, **1i**, **3a**, **4a**, **5a**, **6a**, **7a**, and **8a**, have previously been described as substances possessing cytotoxic, antiviral, and DNA intercalating properties [9,33]. The chemical library comprised eight series of compounds containing diethylamine, 2-methylpiperidine, piperidine, morpholine, 4-methylpiperazine, 4-methylpiperidine, pyrrolidine, and azepane groups attached to the indoloquinoxaline moiety through a dimethylene linker. The presence of these side chains improves the solubility of the drug compound, thereby enhancing its bioavailability [11]. While the core tetracyclic moiety binds tightly to the hinge region deep inside the kinase active site, such groups can also reside at the solvent-exposed interface, acting as a multifunctional “solvating tail”. Furthermore, by interacting with the more variable outer regions of the binding pocket, this group enables the inhibitor to discriminate between highly conserved kinase families; such a discrimination significantly enhances target selectivity [53]. It was recently reported that indolo[2,3-*b*]quinoxaline derivatives containing an alkyne linker, compounds chemically related to those presented in Table 1, exhibit antitumor activity against human ovarian cancer (OVCAR-3) and colon adenocarcinoma (HT-29) cell lines [54]. Moreover, the relative cytotoxicity (determined using human leukemia HL-60 cell lines) of compounds containing an indolo[2,3-*b*]quinoxaline moiety varies significantly depending on the nature of the side chain attached to the nitrogen atom of the indole ring [6].

Planar fused heterocyclic scaffold, the class of compounds to which the molecules in this library belong (see Figure 1a), rank among the most successful “privileged structures” employed for kinase inhibition [15,16,17]. Furthermore, an additional rule of drug-likeness may be applied to kinase-focused screening sets [55]. Thus, the ADME properties of the compounds included in this screening set were preliminarily determined using the SwissADME tool, with the aim of characterizing their physicochemical profiles and verifying their compliance with criteria for drug-like compounds, as well as with kinase-oriented rules of drug-likeness [55]. All the compounds displayed molecular weights below 425, calculated Log P values below 5.3, and no more than six hydrogen-bond acceptors (HBA) or one hydrogen-bond donor (HBD) (Table 2). In addition, every member of the library contained exactly four aromatic rings, meeting the aromatic rings (AR) ≤ 4 criterion without exception. Thus, the presented library of 6-(2-aminoethyl)-6*H*-indolo[2,3-*b*]quinoxalines well satisfies the “kinase-oriented drug-likeness rule”, namely: MW ≤ 500, Log P ≤ 5, HBA ≤ 8, HBD ≤ 3, and AR ≤ 4 [55]. Notably, most of the compounds show no violations of Lipinski’s rule of five; only ten compounds (**2c**, **6c**, **8c**, **2d**, **6d**, **8d**, **2f**, **3f**, **6f**, and **8f**) in the series have a single violation.

### 3.2. Cytotoxic Activity of 6-(2-Aminoethyl)-6H-indolo[2,3-b]quinoxalines

Sixty-five 6-(2-aminoethyl)-6*H*-indolo[2,3-*b*]quinoxalines were evaluated for cytotoxic activity in two human monocyte-like cell lines, THP-1 and MonoMac-6, derived from acute monocytic leukemia AML-5 type. The results of this assay, expressed as IC_50_ values, are presented in Table 3 in comparison with sunitinib, a known FDA-approved anticancer drug that acts as a multi-target RTK inhibitor. Sunitinib inhibits more than 80 kinases, primarily targeting vascular endothelial growth factor receptors (VEGFR1-3), platelet-derived growth factor receptors (PDGFR-α and PDGFR-β), c-Kit (stem cell factor receptor), and two main mutant forms of the FLT3 kinase [56,57]. In this study, the CellTiter-Glo luminescent assay was used to assess cytotoxicity. This assay is based on the quantification of intracellular ATP, a parameter directly proportional to the number of metabolically active, viable cells. A decrease in luminescence signal intensity compared to untreated control cells indicates cytotoxicity of the test compound, cell death, or inhibition of cell proliferation.

**Figure 2:**
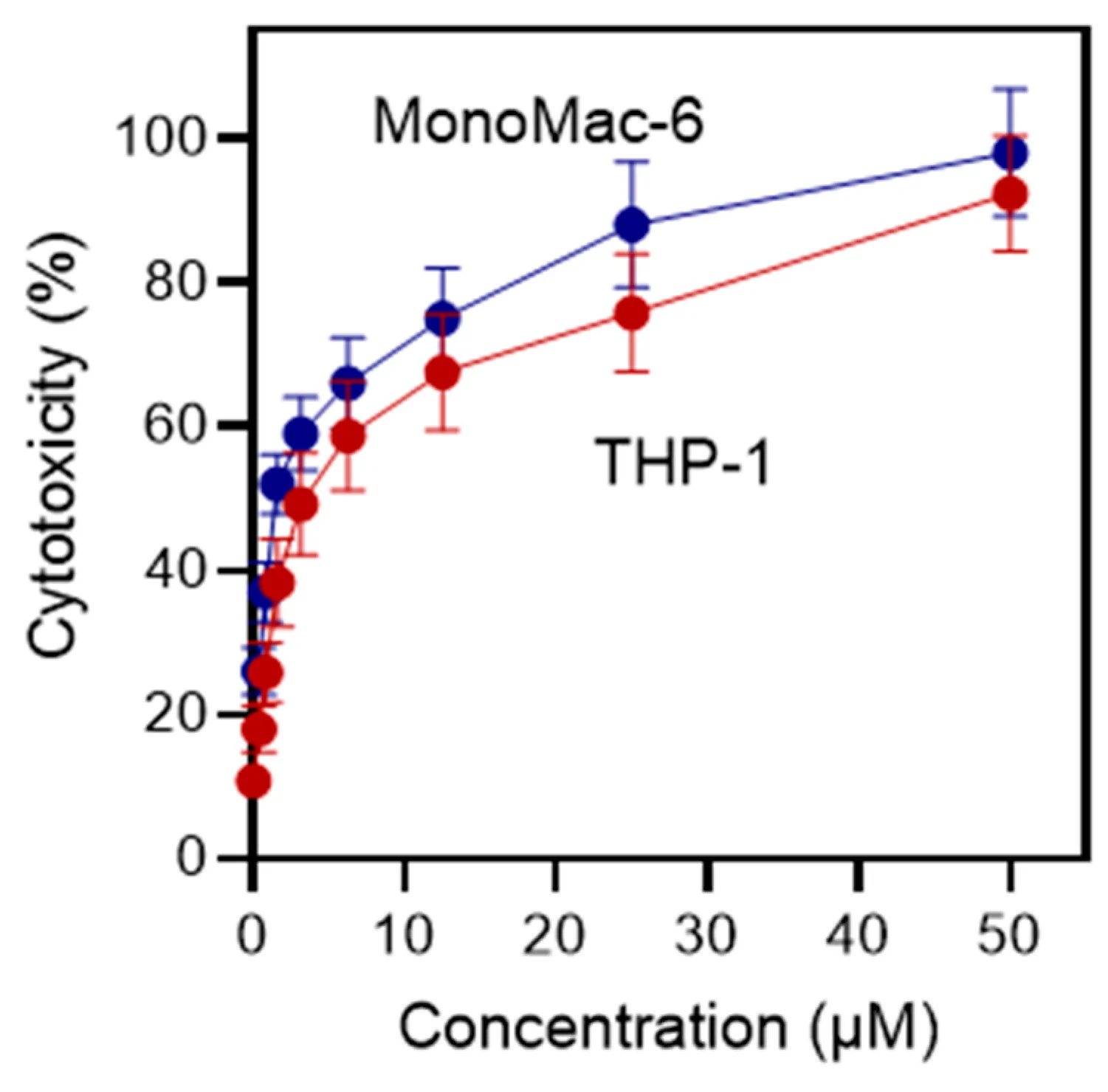
Cytotoxic activity of compound **3g** in THP-1 and MonoMac-6 cells.

Compound **3g** demonstrated relatively high cytotoxicity against THP-1 and MonoMac-6 cells, with IC_50_ values of 3.5 and 1.9 µM, respectively (Table 3). Furthermore, the cytotoxicity of compound **3g** in MonoMac-6 cells was even higher in comparison with the reference compound sunitinib. As an example, Figure 2 illustrates the dose-dependent effect of compound **3g** on the viability of these cells.

Compound **3g**, as well as five other compounds (**1g**, **2g**, **4g**, **7g**, and **8g**) exhibiting an IC_50_ cytotoxicity value <10 µM in THP-1 cells, belong to the class of nitro compounds. Cytotoxic properties of nitro compounds are frequently mediated by the formation of reactive intermediates, including nitro radical anions and subsequent reactive oxygen species (ROS). This process, often referred to as redox cycling, directly damages cellular macromolecules and triggers cell death [58,59]. Although the presence of a nitro group may determine the free-radical mechanisms of action of these compounds, mediated by ROS, the multifaceted nature of the properties of nitro compounds cannot be ruled out [60]. Compounds **1i** and **3h**, containing an oxygen atom in the R^1^ substituent, also exhibited high cytotoxic activity, whereas other compounds bearing a hydroxyl or OMe group demonstrated either low activity or were completely inactive (Table 3). It should be noted that compounds **4a**–**i** containing a morpholine group exhibited lower activity in the cytotoxicity assay (five inactive and three weakly active compounds).

**Figure 3:**
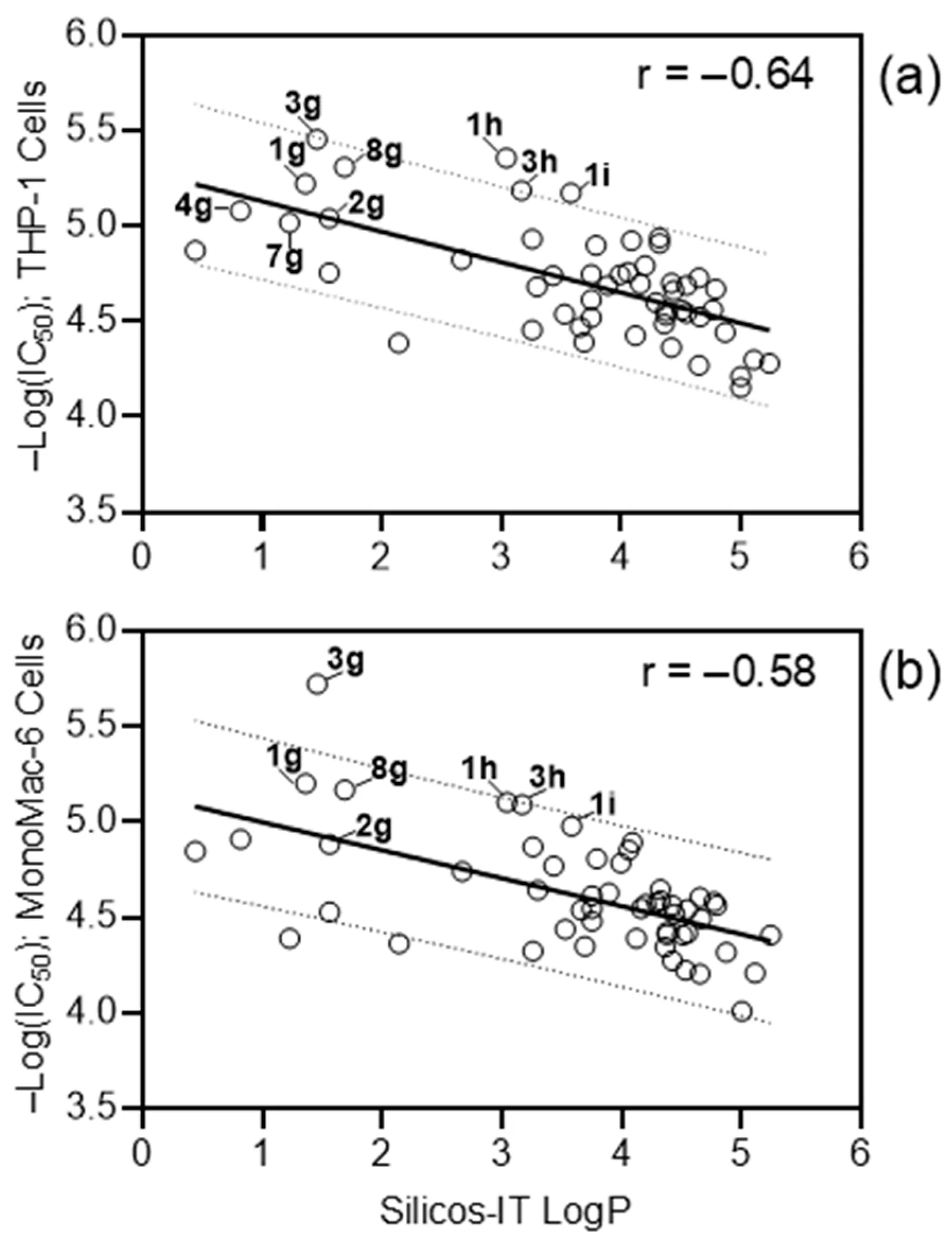
Plot of the cytotoxic activity of sixty-five studied compounds in THP-1 (**a**) and MonoMac-6 (**b**) cells versus lipophilicity. Cytotoxic activity is presented as −Log(IC_50_), and lipophilicity was calculated as Silicos-IT Log P using SwissADME. The dashed lines indicate the area within which 90% of future individual data points are expected to fall. Each compound designation on the panels denotes a compound exhibiting an IC_50_ cytotoxicity value <10 µM in a cellular test system.

To rationalize the experimental cytotoxic potencies of the investigated 6-(2-aminoethyl)-6*H*-indolo[2,3-*b*]quinoxalines, correlation analysis was performed using previously calculated ADME parameters. Among a panel of calculated molecular descriptors (including molecular weight, topological polar surface area, number of rotatable bonds, and various lipophilicity indices), the Silicos-IT Log P descriptor exhibited the most pronounced relationship with the observed cytotoxicity data. Silicos-IT Log P is calculated using a hybrid method relying on 27 fragments and 7 topological descriptors [61], thereby providing a more comprehensive measure of hydrophobic character than simpler atom-based or purely topological models. Consequently, this descriptor was selected for further regression analysis. Although the resulting Pearson linear correlation coefficients (r) were modest (r = −0.64 and −0.58 for compounds tested on THP-1 and MonoMac-6 cells, respectively; Figure 3), the observed trends were statistically significant (*p* < 0.05) for both cell lines, suggesting that cellular permeability governed by overall compound lipophilicity contributes noticeably to the cytotoxic activity of this chemotype. Nevertheless, the modest correlation suggests that factors beyond the lipophilicity of indolo[2,3-*b*]quinoxalines, such as specific target interactions, contribute to the observed cytotoxicity.

### 3.3. Identification of Potential Kinase Targets

Protein kinases play critical, often causative, roles in the cytotoxic activity of many anticancer compounds [62]. Thus, compound **3g**, which demonstrated potent cytotoxic activity in both cell lines, was selected for further kinase profiling in a competition binding assay for its ability to compete with an active-site-directed ligand for 97 different kinases representing all known kinase families (Eurofins DiscoverX scanEDGE^®^). Compound **3g** was screened at 10 μM, and the kinases for which ≥65% inhibition of ligand binding was observed were designated as “kinase targets of the compound”. Ten such kinase targets were identified, including seven kinases belonging to the class of tyrosine kinases or tyrosine kinase-like (TKL) kinases (Table 4). The complete results of this screening are presented in Table S1.

Because tyrosine kinase inhibitors can exhibit direct cytotoxic activity in cancer cells by inducing apoptosis and disrupting crucial cell survival and proliferation pathways [63,64], the compound **3g** was also profiled in a competition binding assay for its ability to compete with different tyrosine kinases (Eurofins DiscoverX scanTK™ panel). This subset comprises 135 distinct receptor and non-RTKs, including critically important mutant forms associated with various human diseases. The complete results of this screening are presented in Table S2. Compound **3g** exhibits relatively high binding activity against fourteen of the studied tyrosine kinases, with a ligand binding inhibition level of ≥65%. These kinases included TYK2, ZAP70, eight mutant forms of ABL1, two mutant forms of FLT3, and one mutant form of ALK. The compound has the highest binding activity against one of the mutated forms of FLT3 kinase, FLT3 (D835V) (Table 5).

It should be noted that compound **3g** also demonstrated relatively high binding selectivity for non-mutant kinases during screening on the scanEDGE and scanTK panels (Table 6). The selectivity score (S-score) was calculated using “percent of control” as a potency threshold and provides a quantitative method of describing compound selectivity to facilitate comparison of different compounds [65].

The ability to bind to multiple mutated forms of ABL1 may be of interest for further research. ABL1 is a non-RTK that plays a role in many key processes linked to cell growth and survival, such as cytoskeleton remodeling in response to extracellular stimuli, cell motility and adhesion, receptor endocytosis, autophagy, DNA damage response and apoptosis [66].

New FLT3 inhibitors with high affinity for various mutant forms of FLT3, specifically the D835V mutation, have recently been described [67]. The authors employed the same methodology used in the present study: cell-free scanEDGE and scanELECT assays from Eurofins/DiscoverX. Among these inhibitors, the compound 4-(*tert*-butyl)-*N*-(3-phenyl-1*H*-pyrazol-5-yl)benzamide (compound **1247** in [67]) demonstrated the highest affinity (>99% at a concentration of 10 µM) for several mutant forms of FLT3, including FLT3-D835V. However, this compound also exhibited significant binding to the non-mutant (wild-type) form of FLT3.

Since mutations in the FLT3 gene are relatively common in AML, activating mutations in FLT3 represent an appealing target for drug development [68,69]. Thus, we also evaluated the binding affinity of compound **3g** and a series of its analogs (predominantly with a nitro group at the R^1^ position) for the mutant form FLT3 (D835V), as primary screening revealed the highest binding activity specifically against this mutant form of FLT3 (see Table 5). Based on the results of secondary screening, compound **3h** (9-hydroxy-6-(2-(piperidin-1-yl)ethyl)-6*H*-indolo[2,3-*b*]quinoxaline) demonstrated the highest binding affinity, with the K_d_ value falling within the nanomolar range (Table 7).

The limited set of compounds tested against the FLT3 (D835V) kinase precludes the formulation of definitive structure-activity relationships (SAR). Nevertheless, the obtained results already demonstrate that substituents R and R^1^ (see Table 1) can exert a significant influence on this activity. For example, compounds **4g** and **8g**, bearing morpholine and azepane substituents at position R, are inactive, while relatively high activity was observed for compound **2g**, which contains a 2-methylpiperidine group at this position. The inability of compound **4g** to bind to the kinase may be attributed to the presence of an electronegative oxygen atom in the morpholine structure; furthermore, morpholine has additional possibilities in the formation of hydrogen bonds, which affect its binding to biotargets.

### 3.4. Molecular Docking Studies

The DFG pocket is a transient, hydrophobic, allosteric binding site in protein kinases created when the conserved Asp-Phe-Gly (DFG) motif at the start of the activation loop flips “out” (DFG-out) from the active site, inactivating the enzyme [70]. It is a critical target for type II kinase inhibitors, which lock the kinase in this inactive conformation [70,71,72]. To rationalize the observed experimental binding preferences of 6-(2-aminoethyl)-6*H*-indolo[2,3-*b*]quinoxalines to mutated FLT3 (D835V), molecular docking was performed with compounds **3g** and **3h** into the ATP-binding sites of two FLT3 structures: 4RT7 (co-crystallized with the type II inhibitor quizartinib, representing the inactive DFG-out conformation) and 6JQR (co-crystallized with the type I inhibitor gilteritinib, representing the active DFG-in conformation). The 6JQR structure lacks residues 831–836 in the activation loop. Therefore, this segment was modeled using the active conformation of the closely related KIT kinase (PDB 1PKG, 56% sequence identity to the FLT3 kinase domain) as a template via SWISS-MODEL [73], following the protocol used by Wang et al. [39]. D835V mutants of both structures were also prepared, with local energy minimization applied to all residues within 8 Å of the mutated site to relieve steric clashes and propagate conformational effects into the binding pocket. The obtained docking scores and key interactions are presented in Table 8.

**Figure 4:**
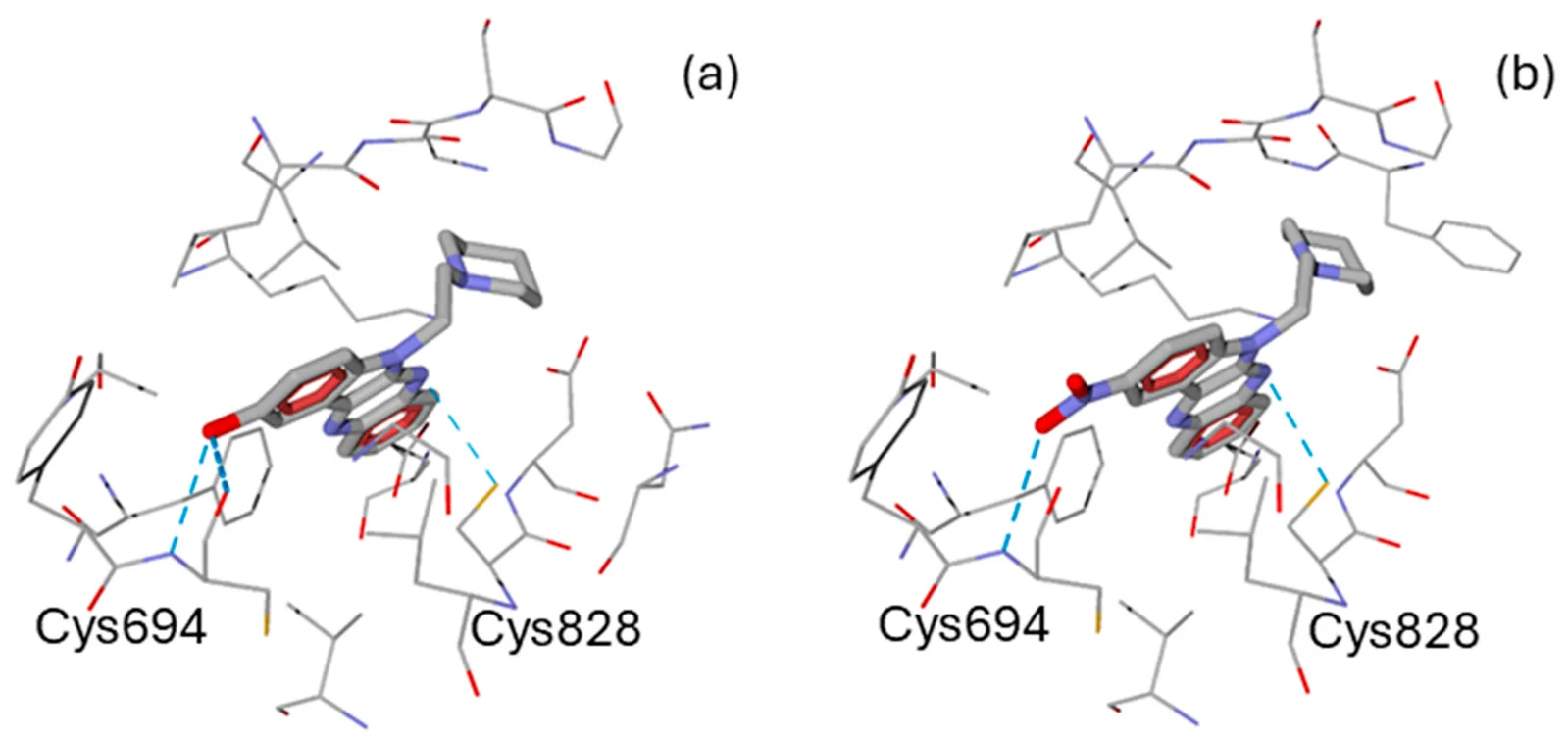
The docking poses of compounds **3h** (**a**) and **3g** (**b**) in the D835V mutant of FLT3 (DFG-in conformation). Residues within 3 Å of the pose are visible. Hydrogen bonds are shown in blue dashed lines.

Both compounds **3h** and **3g** produced sufficiently negative DS values across all systems, consistent with favorable binding poses anchored by hydrogen bonds involving the hydroxy/nitro substituents and the pyrazine nitrogen (Table 8, Figure 4). However, clear conformational and mutational trends should be noted. DS values were markedly more favorable (more negative) for the 6JQR-based (active, DFG-in) structures than for 4RT7 (inactive, DFG-out), with the greatest affinity observed for the 6JQR (D835V) models (Table 8). The D835V mutation further improved scores relative to the corresponding wild-type structures, even though the mutated residue lies distal to the orthosteric site. Local optimization after mutagenesis altered side-chain orientations of several binding-site residues, including those in the hinge and DFG regions, demonstrating long-range conformational coupling.

### 3.5. MD Simulation

**Figure 5:**
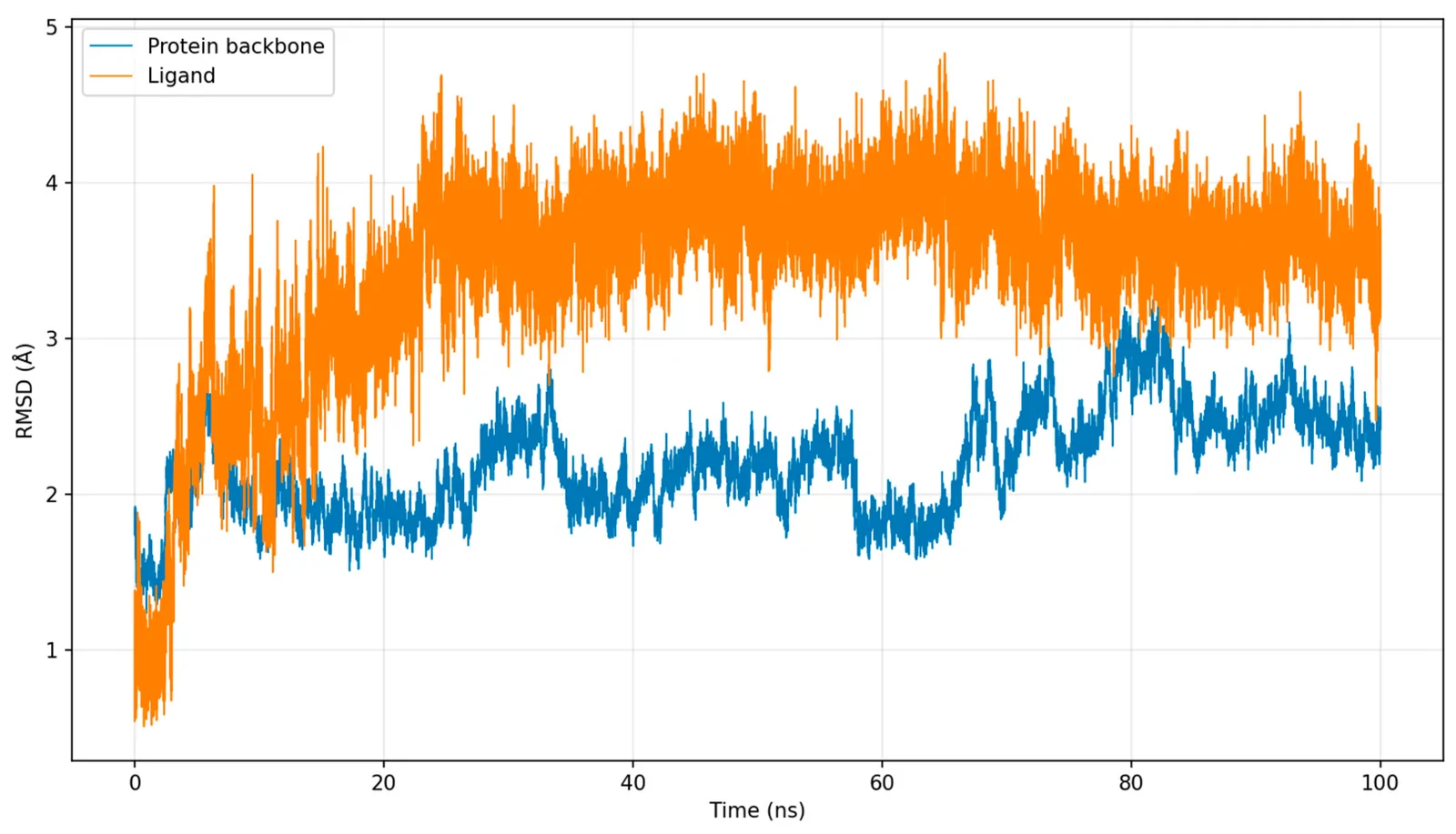
Time evolution of the RMSD of atoms in the protein backbone and heavy atoms of **3h** during the MD simulation with NAMD 3.1.

To evaluate binding stability, an MD simulation was performed. The structural stability of the FLT3 (D835V) in complex with **3h** was evaluated from the time evolution of the root-mean-square deviation (RMSD) of the protein backbone and the heavy-atom RMSD of the ligand, computed against the energy-minimized starting structure. The protein backbone came to equilibrium within the first 10–15 ns (Figure 5) and subsequently fluctuated within a small range with a mean Cα RMSD of 2.19 ± 0.33 Å (minimum 1.24 Å, maximum 3.25 Å). The RMSD of **3h** has a higher mean value of 3.45 ± 0.66 Å (minimum 0.51 Å, maximum 5.86 Å). We attribute this difference to the modular architecture of the ligand rather than any partial dissociation event. In molecule **3h**, the condensed tetracyclic indolo[2,3-*b*]quinoxaline core, which possesses a planar geometry, is linked via a flexible ethyl linker to a saturated, conformationally mobile piperidine ring. The rigid aromatic core inserts into the hydrophobic adenine sub-pocket of the kinase, where it is locked by aromatic and van der Waals contacts, while the piperidinyl-ethyl arm protrudes from the ATP cleft toward the solvent-exposed front pocket. Because the ligand RMSD is dominated by atomic displacements of the most peripheral substituents, even small dihedral rearrangements of the ethyl bridge inflate the global RMSD by 1–2 Å without any displacement of the core from its binding site.

The most persistent hydrogen bond is formed between the backbone carbonyl of Cys694 and the ligand proton of the hydroxyl group (this bond was found in 16% of MD frames). A less stable hydrogen bond (12% of MD frames) was found between the backbone carbonyl of Cys694 and the proton of carbon in the 10th position of the indoloquinoxaline scaffold (Figure S1). This hinge contact is similar to crenolanib [39], though with less stable bonding, provides a rational structural basis for the biological activity of the compound against the D835V resistance mutant: because D835V is a distal activation-loop mutation that does not perturb the hinge backbone geometry directly, an inhibitor whose principal pharmacophoric anchor coincides with the canonical Cys694 contact is intrinsically less susceptible to this class of resistance, in line with the type-I-like behavior of crenolanib that retains activity against D835V/Y/H mutants [74]. It should be noted that hydrogen bonding of compound **3h** with the Cys694 residue is also among the important interactions of the initial docking pose (Table 8).

The thermodynamic favorability of **3h** binding was quantified by single-trajectory MM-GBSA analysis [75]. The binding free energy obtained for the **3h**/FLT3(D835V) complex is ΔG = −31.64 ± 4.13 kcal/mol. This value consists of several types of interactions (Table 9). Most notably, the van der Waals contacts account for the overwhelming majority of the favorable interaction energy, which is typical of this type of kinase [39].

These computational results present the structural basis for the experimentally observed preference of the indoloquinoxaline series for FLT3 (D835V). The D835 residue normally stabilizes an inactive kinase conformation through hydrogen bonding that maintains a short α-helical segment in the activation loop. Replacement by valine eliminates these bonds, introduces steric bulk, and shifts the equilibrium toward the active DFG-in state [39]. Consequently, type II inhibitors that require the DFG-out pocket (exemplified by quizartinib in 4RT7) lose potency against D835 mutants, whereas type I inhibitors that engage the DFG-in conformation (exemplified by gilteritinib in 6JQR) retain or gain activity [25,76]. The present docking poses place the planar indoloquinoxaline core in the adenine pocket, with the hydroxy or nitro groups forming hinge-region hydrogen bonds (particularly with Cys694 in the 6JQR series) and the pyrazine nitrogen interacting near Cys828. These interactions are strengthened in the DFG-in mutant models, where Phe830 reorientation creates a more accommodating hydrophobic environment. The superior DS and interaction profile of **3h** versus **3g** in the 6JQR (D835V) system correlates directly with its lower experimental K_d_ (410 nM).

The persistence of at least one intermolecular hydrogen bond and of the Cys694 anchor in particular throughout the production trajectory, taken together with the stable RMSD plateau, the bounded ligand RMSD, and the dispersion-dominated MM-GBSA profile, collectively support the conclusion that molecule **3h** forms a stable, tightly bound, type-I-like complex with the active DFG-in conformation of FLT3 (D835V) kinase. Thus, the MD simulation results corroborate the docking and binding data by showing that compound **3h** remains stably accommodated in the ATP-binding site of FLT3 (D835V).

It should be noted that identifying more effective inhibitors of mutant tyrosine kinase forms (including FLT3-D835V) requires a second screening stage; this involves testing on specific cell lines harboring the mutation and validating the results using wild-type cell lines. The high affinity of compound **3h** for the mutant form of the FLT kinase, combined with the high cytotoxic activity of compound **1h** (Table 3), suggests that further synthesis and investigation of compounds containing a hydroxyl group at the R^1^ position is warranted. On the other hand, in addition to the cytotoxic activity of quinoxalines, driven by the presence of a nitro group (i.e., a redox-dependent mechanism of action) and their high affinity for tyrosine kinases, these compounds may have other targets. In particular, quinoxaline-based histone deacetylase (HDAC) inhibitors have been described [77,78]. These inhibitors belong to a diverse group of compounds that block enzymes responsible for removing acetyl groups from histones and non-histone proteins. Such action alters gene transcription, induces cell cycle arrest and stimulates apoptosis, making them key agents in cancer therapy.

## 4. Conclusions

Based on screening results using two kinase panels, compound **3g** exhibits relatively high binding activity against tyrosine kinases TYK2, ZAP70, eight mutant forms of ABL1, two mutant forms of FLT3, and one mutant form of ALK, and also demonstrates relatively high binding selectivity towards non-mutant tyrosine kinases. Data on direct binding affinity and computational modeling results confirm that 6-(2-aminoethyl)-6*H*-indolo[2,3-*b*]quinoxalines **3g** and **3h** selectively target the active, D835-mutant form of FLT3. Consequently, this structural scaffold represents a promising starting point for the development of novel targeted therapeutics directed against mutant forms of FLT3, particularly in cases where D835-type mutations predominate. These findings suggest that compound **3h** serves as a promising lead structure for the further optimization of type I FLT3 (D835V) inhibitors.

## Figures and Tables

**Table 1:** Chemical structures of 6-(2-aminoethyl)-6*H*-indolo[2,3-*b*]quinoxalines in the screening library.

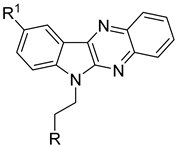									
R	R^1^								
	H	Et	*i*-Pr	Br	Cl	*t*-Bu	NO_2_	HO	MeO
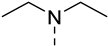	**1a**	**1b**	**1c**	**1d**	**1e**	**1f**	**1g**	**1h**	**1i**
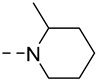	**2a**	**2b**	**2c**	**2d**	**2e**	**2f**	**2g**		
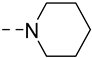	**3a**	**3b**	**3c**	**3d**	**3e**	**3f**	**3g**	**3h**	
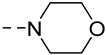	**4a**	**4b**	**4c**	**4d**	**4e**	**4f**	**4g**	**4h**	**4i**
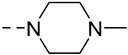	**5a**	**5b**	**5c**	**5d**	**5e**	**5f**	**5g**	**5h**	**5i**
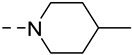	**6a**	**6b**	**6c**	**6d**	**6e**	**6f**	**6g**	**6h**	**6i**
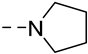	**7a**	**7b**	**7c**	**7d**	**7e**	**7f**	**7g**		
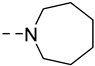	**8a**	**8b**	**8c**	**8d**	**8e**	**8f**	**8g**		

**Table 2:** A summary of general molecular properties of approved protein kinase inhibitors and compounds in the present screening set.

Set or Rules Applied	MW (Mean)	Log P (Mean)	HBA (Mean)	HBD (Mean)	AR (Mean)
Kinase inhibitors in clinical development ^a^	≤615.8 (453.3)	≤7.01 (3.7)	≤10 (5.7)	≤4 (1.9)	≤5 (3.2)
Rules applied for virtual screening	≤500	≤5	≤8	≤3	≤4
Present screening set	<425 (372.7)	<5.3 (3.70) ^b^	≤6 (3.66)	≤1	=4

^a^ A total of 52 kinase inhibitors (12 approved and 40 in clinical development) were used for the calculations of the parameters by Li et al. [55]. ^b^ The Silicos-IT Log P values were used. Abbreviations: MW, molecular weight; Log P, calculated Log P value; HBA, number of hydrogen bond acceptors; HBD, number of hydrogen bond donors; AR, number of aromatic rings.

**Table 3:** Cytotoxic activity of 6-(2-aminoethyl)-6*H*-indolo[2,3-*b*]quinoxalines in THP-1 and MonoMac-6 cells.

Compound	Cytotoxicity (IC_50_, µM)		Compound	Cytotoxicity (IC_50_, µM)	
	THP-1	MonoMac-6		THP-1	MonoMac-6
**1a**	N.T.	N.T.	**5a**	N.T.	N.T.
**1b**	21.7 ± 3.7	30.3 ± 5.2	**5b**	29.0 ± 4.2	36.4 ± 4.5
**1c**	29.9 ± 3.8	32.2 ± 4.5	**5c**	17.9 ± 3.9	28.4 ± 6.2
**1d**	16.1 ± 3.7	26.9 ± 3.8	**5d**	20.8 ± 4.4	22.8 ± 4.7
**1e**	20.1 ± 4.0	28.4 ± 4.1	**5e**	35.1 ± 5.5	47.5 ± 5.3
**1f**	N.T.	N.T.	**5f**	18.0 ± 4.8	16.4 ± 3.3
**1g**	6.0 ± 1.7	6.3 ± 1.6	**5g**	13.4 ± 2.3	14.2 ± 2.8
**1h**	4.4 ± 1.4	7.9 ± 2.6	**5h**	41.0 ± 4.8	43.4 ± 5.8
**1i**	6.7 ± 1.2	10.5 ± 2.8	**5i**	15.0 ± 2.2	18.2 ± 3.9
**2a**	24.3 ± 3.3	33.1 ± 5.1	**6a**	30.2 ± 4.8	24.3 ± 3.4
**2b**	18.7 ± 3.2	24.8 ± 4.9	**6b**	53.7 ± 5.7	62.4 ± 7.4
**2c**	36.1 ± 5.3	48.1 ± 5.1	**6c**	N.T.	N.T.
**2d**	19.9 ± 4.3	27.3 ± 5.8	**6d**	43.2 ± 5.9	53.1 ± 6.3
**2e**	29.3 ± 5.3	37.7 ± 4.4	**6e**	27.9 ± 5.9	38.9 ± 4.9
**2f**	N.T.	N.T.	**6f**	50.2 ± 6.8	61.7 ± 7.7
**2g**	9.1 ± 1.9	29.6 ± 4.7	**6g**	17.6 ± 4.3	13.1 ± 2.6
**3a**	33.9 ± 4.2	29.0 ± 4.7	**6h**	11.7 ± 2.7	13.5 ± 3.7
**3b**	20.5 ± 3.8	28.6 ± 5.2	**6i**	12.6 ± 1.8	15.6 ± 3.3
**3c**	N.T.	N.T.	**7a**	18.2 ± 2.5	17.0 ± 3.2
**3d**	11.5 ± 3.3	22.5 ± 4.4	**7b**	12.3 ± 3.6	25.8 ± 3.9
**3e**	25.2 ± 4.3	26.5 ± 4.6	**7c**	N.T.	59.6 ± 7.1
**3f**	70.2 ± 6.2	N.T.	**7d**	11.9 ± 2.4	12.8 ± 3.3
**3g**	3.5 ± 0.8	1.9 ± 0.4	**7e**	17.5 ± 3.9	14.0 ± 2.8
**3h**	6.5 ± 1.6	8.1 ± 3.0	**7f**	27.4 ± 5.8	26.2 ± 5.2
**4a**	N.T.	N.T.	**7g**	9.6 ± 2.6	40.7 ± 5.8
**4b**	N.T.	N.T.	**8a**	20.5 ± 3.4	23.6 ± 4.5
**4c**	37.5 ± 4.4	40.7 ± 5.3	**8b**	21.4 ± 4.5	27.3 ± 3.8
**4d**	40.6 ± 6.1	44.7 ± 4.9	**8c**	61.6 ± 7.3	98.0 ± 10.6
**4e**	N.T.	N.T.	**8d**	28.6 ± 5.3	38.2 ± 4.9
**4f**	32.7 ± 5.1	45.3 ± 5.7	**8e**	27.6 ± 4.8	39.2 ± 5.5
**4g**	8.3 ± 1.8	12.3 ± 2.9	**8f**	52.5 ± 6.7	39.1 ± 5.2
**4h**	N.T.	N.T.	**8g**	4.9 ± 1.4	6.8 ± 2.2
**4i**	N.T.	N.T.	Sunitinib	14.9 ± 0.9	5.7 ± 0.3

Abbreviations: N.T., no cytotoxicity at concentrations ≤ 100 μM.

**Table 4:** Selected kinase targets of compound **3g** with a ligand binding inhibition (inhib.) level of ≥65%, identified during Eurofins DiscoverX scanEDGE kinase profiling.

Kinase Name	Abbreviation	Kinase Class/Group	Inhib. (%) ^a^
Abelson proto-oncogene 1 (E255K)-P	ABL1 (E255K)	Non-RTK	68
Anaplastic lymphoma kinase	ALK	RTK	84
Bone morphogenetic protein receptor 2	BMPR2	Serine/threonine kinase, TKL	65
B-Raf proto-oncogene	BRAF (V600E)	Serine/threonine kinase, TKL	65
Inhibitor of nuclear factor κ-B kinase α	IKK-α	Serine/threonine kinase, CAMK	66
Platelet-derived growth factor receptor α	PDGFRA	RTK	66
PI-4,5-bisphosphate 3-kinase, subunit α	PIK3CA	Lipid kinase	68
PI-4,5-bisphosphate 3-kinase, subunit γ	PIK3CG	Lipid kinase	74
Tyrosine kinase 2	TYK2	Non-RTK	72
Zeta-chain-associated protein kinase 70	ZAP70	Non-RTK	65

^a^ Percentage inhibition of binding to an active-site-directed ligand for each of the indicated kinases after treatment with 10 μM compound **3g**. Abbreviations: CAMK, calcium/calmodulin-dependent protein kinase; PI, phosphatidylinositol; TKL, tyrosine kinase-like.

**Table 5:** Selected kinase targets of compound **3g** with a ligand binding inhibition level of ≥65%, identified during Eurofins DiscoverX scanTK™ panel profiling.

Abbreviation	Inhibition (%) ^a^	Abbreviation	Inhibition (%)
ABL1 (F317I)-non-P	71	FLT3 (D835Y)	25
ABL1 (H396P)-non-P	77	FLT3 (K663Q)	0
ABL1 (H396P)-P	71	FLT3 (R834Q)	69
ABL1 (M351T)-P	67	FLT3 (N841I)	6
ABL1 (Q252H)-P	72	FLT3 (ITD)	0
ABL1 (T315I)-non-P	66	FLT3 (ITD, D835V)	60
ABL1 (T315I)-P	68	FLT3 (ITD, F691L)	65
ABL1 (Y253F)-P	69	FLT3-autoinhib.	43
ALK (C1156Y)	76	KIT (D816H)	71
FLT3	0	TYK2 (JH1 dom. cat.)	74
FLT3 (D835H)	8	ZAP70	76
FLT3 (D835V)	99		

^a^ Percentage inhibition of binding to an active-site-directed ligand for each of the indicated kinases after treatment with 10 μM compound **3g**. Abbreviations: ABL, Abelson proto-oncogene 1; ALK, anaplastic lymphoma kinase; FLT3, FMS-like tyrosine kinase 3; KIT, proto-oncogene c-Kit; TYK2, Tyrosine kinase 2; ZAP70, Zeta-chain-associated protein kinase 70.

**Table 6:** Binding selectivity of compound **3g** at tested 10 µM concentration using Eurofins DiscoverX scanEDGE^®^ and scanTK™ panels.

Panel of Kinases	Threshold Range	N	n	S-Score
scanEDGE^®^	S(35)	6	90	0.067
	S(10)	0	90	0
	S(1)	0	90	0
scanTK™	S(35)	2	83	0.024
	S(10)	0	83	0
	S(1)	0	83	0

Selectivity score (S-score) is calculated by dividing the number of kinases (N) that compounds bind to by the total number (n) of distinct kinases tested, excluding mutant variants. S(35), S(10), and S(1) are values, expressed as (number of non-mutant kinases with “percent of control” <35%, <10, or <1%, respectively)/(number of non-mutant kinases tested).

**Table 7:** Binding affinity of selected compounds toward mutated FLT3 (D835V).

Compound	K_d_ (µM)	Compound	K_d_ (µM)
**1g**	8.8 ± 0.5	**5g**	25.0 ± 2.3
**2g**	0.82 ± 0.09	**6h**	2.2 ± 0.3
**3g**	11.0 ± 0.6	**7e**	2.2 ± 0.3
**3h**	0.41 ± 0.07	**7g**	3.9 ± 0.4
**4g**	N.A.	**8g**	N.A.

N.A., no FLT3 (D835V) binding affinity at concentrations ≤ 30 μM.

**Table 8:** Docking scores (DS) and key hydrogen bonding interactions of compounds **3h** and **3g** with FLT3 structures and D835V mutants.

Compound	DS ^a^	Hydrogen Bonding Interactions ^b^
4RT7; DFG-out Conformation		
**3h**	−103.46	Tyr693 (OH)
**3g**	−104.20	Lys614 (NO_2_), Tyr693 (NO_2_, weak)
4RT7 (D835V); DFG-out Conformation		
**3h**	−123.68	Asp829 (pyrazine, weak)
**3g**	−120.89	-
6JQR; DFG-in Conformation		
**3h**	−120.28	Lys644 (OH), Glu661 (OH)
**3g**	−121.26	Phe621 (NO_2_), Gly622 (NO_2_), Lys644 (NO_2_), Lys644 (pyrazine), Cys828 (pyrazine)
6JQR (D835V); DFG-in Conformation ^c^		
**3h**	−135.59	Cys694 (OH, two bonds), Cys828 (pyrazine, weak)
**3g**	−131.97	Cys694 (NO_2_), Cys828 (pyrazine)

^a^ A unitless measure calculated with the MolDock force field [40]. ^b^ Ligand groups participating in the hydrogen bond formation are given in parentheses. ^c^ Partial docking scores of individual amino acid residues for the poses of compounds **3h** and **3g** in 6JQR (D835V) (DFG-in conformation) are presented in Table S3.

**Table 9:** Composition of binding free energies obtained on the **3h**/FLT3(D835V) complex simulation.

Type of Intermolecular Interactions and Geometric/Thermodynamic Metrics	Energy (kcal/mol)
Van der Waals Interactions	−44.65
Electrostatics (Coulomb Interactions)	−9.55
Generalized Born Solvation ^a^	28.03
Solvent Accessible Surface Area (SASA) ^b^	−5.47
ΔG total (estimated binding free energy)	−31.64

^a^ Energy cost of desolvating both partners upon binding. ^b^ Energy gain from burying nonpolar surface.

## Data Availability

Data are contained within the article.

## Supplementary Materials

The following supporting information can be downloaded at: https://www.mdpi.com/article/10.3390/biom16081173/s1, Table S1: Kinase profile of compound **3g**. The kinases were evaluated using the Eurofins DiscoverX scanEDGE^®^ panel, as described under Materials and Methods. Shown is the percentage inhibition of binding to an active-site-directed ligand for each of the indicated kinases after treatment with 10 μM of compound **3g**; Table S2: Tyrosine kinase profile of compound **3g**. The kinases were evaluated using the Eurofins DiscoverX scanTK™ panel, as described under Materials and Methods. Shown is the percentage inhibition of binding to an active-site-directed ligand for each of the indicated kinases after treatment with 10 μM of compound **3g**; Table S3: Partial docking scores of ten highly interacting residues for the docking poses of compounds **3h** and **3g** in 6JQR (D835V) structure (DFG-in conformation); Figure S1: MD-predicted orientation of compound **3h** in the ATP-binding site of D835V mutant FLT3. Purple dashed lines represent the most persistent hydrogen bonds.
