# Supplementary material for: 6-(2-Aminoethyl)-6H-indolo[2,3-b]quinoxalines as Promising Compounds Capable of Binding to FLT3 (D835V) Kinase
Source: Biomolecules. 2026 Aug 12;16(8):1173. doi: 10.3390/biom16081173 (PMC13511512; doi:10.3390/biom16081173)
Supplement: Supplementary file 1 [file biomolecules-16-01173-s001.zip › biomolecules-4466485-supplementary.pdf]

**Supplementary Materials**

**for**

# 6-(2-Aminoethyl)-6*H*-indolo[2,3-*b*]quinoxalines as Promising Compounds Capable of Binding to FLT3 (D835V) Kinase


**Igor A. Schepetkin[1], Alexander V. Uvarov[2], Egor A. Evriinov[2], Andrei I. Khlebnikov[2]**

**[1]Department of Microbiology and Cell Biology, Montana State University, Bozeman, MT 59717, USA**
**[2]Kizhner Research Center, Tomsk Polytechnic University, Tomsk 634050, Russia**

**Supplementary Table S1**. Kinase profile of compound **3g.** The kinases were evaluated using the Eurofins DiscoverX scanEDGE® panel, as described under Materials and Methods. Shown is the percentage inhibition of binding to an active-site directed ligand for each of the indicated kinases after treatment with 10 μM of compound **3g**.

| Kinase Abbreviation | Inhib. (%) | Kinase Abbreviation | Inhibition (%) |
|---|---|---|---|
| ABL1 (E255K)-P | **68** | LKB1 | 9 |
| ABL1(T315I)-P | 32 | MAP3K4 | 3 |
| ABL1-non-P | 56 | MAPKAPK2 | 3 |
| ABL1-P | 35 | MARK3 | 3 |
| ACVR1B | 4 | MEK1 | 56 |
| ADCK3 | 11 | MEK2 | 52 |
| AKT1 | 7 | MET | 5 |
| AKT2 | 3 | MKNK1 | 22 |
| ALK | **84** | MKNK2 | 52 |
| AURKA | 50 | MLK1 | 3 |
| AURKB | 49 | p38-α | 0 |
| AXL | 0 | p38-β | 6 |
| BMPR2 | **65** | PAK1 | 31 |
| BRAF | 32 | PAK2 | 17 |
| BRAF (V600E) | **65** | PAK4 | 12 |
| BTK | 43 | PCTK1 | 62 |
| CDK11 | 11 | PDGFRA | **66** |
| CDK2 | 2 | PDGFRB | 29 |
| CDK3 | 11 | PDPK1 | 21 |
| CDK7 | 60 | PIK3C2B | 55 |
| CDK9 | 0 | PIK3CA | **68** |
| CHEK1 | 25 | PIK3CG | **74** |
| CSF1R | 1 | PIM1 | 17 |
| CSNK1D | 4 | PIM2 | 8 |
| CSNK1G2 | 11 | PIM3 | 16 |
| DCAMKL1 | 61 | PKAC-α | 6 |
| DYRK1B | 8 | PLK1 | 18 |
| EGFR | 29 | PLK3 | 27 |
| EGFR (L858R) | 35 | PLK4 | 64 |
| EPHA2 | 5 | PRKCE | 49 |
| ERBB2 | 23 | RAF1 | 0 |
| ERBB4 | 5 | RET | 3 |
| ERK1 | 5 | RIOK2 | 31 |
| FAK | 2 | ROCK2 | 43 |
| FGFR2 | 12 | RSK2 (Kin. Dom.; 1-N-term.) | 55 |
| FGFR3 | 17 | SNARK | 60 |

**Supplementary Table S1 (continued)**

| Kinase Abbreviation | Inhib. (%) | Kinase Abbreviation | Inhibition (%) |
|---|---|---|---|
| FLT3 | 2 | SRC | 20 |
| GSK3B | 55 | SRPK3 | 9 |
| IGF1R | 6 | TGFBR1 | 0 |
| IKK-$\alpha$ | **66** | TIE2 | 8 |
| IKK-β | 36 | TRKA | 25 |
| INSR | 54 | TSSK1B | 11 |
| JAK2 (JH1 dom. cat.) | 48 | TYK2 (JH1 dom. cat.) | **72** |
| JAK3 (JH1 dom. cat.) | 59 | ULK2 | 44 |
| JNK1 | 52 | VEGFR2 | 60 |
| JNK2 | 59 | YANK3 | 6 |
| JNK3 | 32 | ZAP70 | **65** |
| KIT | 20 | | |
| KIT (D816V) | 8 | | |
| KIT (V559D, T670I) | 3 | | |

Abbreviations: P, phosphorylated form; non-P, non-phosphorylated form; cat., catalytic; term., terminal; kin., kinase; dom., domain. Residues are indicated in brackets for mutated forms. The percentage of binding inhibition for the 10 kinases for which ≥65% inhibition of ligand binding was observed is shown in bold.

**Supplementary Table S2**. Tyrosine kinase profile of compound **3g.** The kinases were evaluated using the Eurofins DiscoverX scanTK™ panel, as described under Materials and Methods. Shown is the percentage inhibition of binding to an active-site directed ligand for each of the indicated kinases after treatment with 10 µM of compound **3g.**

| **Kinase Abbreviation** | % | **Kinase Abbreviation** | % | **Kinase Abbreviation** | % |
|---|---|---|---|---|---|
| ABL1 (E255K)-P | 55 | EPHA4 | 6 | JAK3 (JH1 dom. cat.) | **65** |
| ABL1 (F317I)-non-P | **71** | EPHA5 | 9 | KIT | 15 |
| ABL1 (F317I)-P | 55 | EPHA6 | 15 | KIT (A829P) | 36 |
| ABL1 (F317L)-non-P | 63 | EPHA7 | 11 | KIT (D816H) | **71** |
| ABL1 (F317L)-P | 55 | EPHA8 | 2 | KIT (D816V) | 7 |
| ABL1 (H396P)-non-P | **77** | EPHB1 | 23 | KIT (L576P) | 17 |
| ABL1 (H396P)-P | **71** | EPHB2 | 9 | KIT (V559D) | 23 |
| ABL1 (M351T)-P | **67** | EPHB3 | 4 | KIT (V559D, T670I) | 15 |
| ABL1 (Q252H)-non-P | 37 | EPHB4 | 13 | KIT (V559D, V654A) | 12 |
| ABL1 (Q252H)-P | **72** | EPHB6 | 64 | KIT-autoinhib. | 40 |
| ABL1 (T315I)-non-P | **66** | ERBB2 | 36 | LCK | 24 |
| ABL1 (T315I)-P | **68** | ERBB3 | 61 | LTK | 10 |
| ABL1 (Y253F)-P | **69** | ERBB4 | 4 | LYN | 0 |
| ABL1-non-P | 51 | FAK | 0 | MERTK | 5 |
| ABL1-P | 45 | FER | 4 | MET | 0 |
| ABL2 | 28 | FES | 11 | MET (M1250T) | 11 |
| ALK | 45 | FGFR1 | 26 | MET (Y1235D) | 0 |
| ALK (C1156Y) | **76** | FGFR2 | 15 | MST1R | 23 |
| ALK (L1196M) | 32 | FGFR3 | 9 | MUSK | 0 |
| AXL | 22 | FGFR3 (G697C) | 25 | PDGFRA | 51 |
| BLK | 23 | FGFR4 | 5 | PDGFRB | 6 |
| BMX | 22 | FGR | 12 | PYK2 | 25 |
| BRK | 15 | FLT1 | 20 | RET | 9 |
| BTK | 0 | FLT3 | 0 | RET (M918T) | 14 |
| CSF1R | 2 | FLT3 (D835H) | 8 | RET (V804L) | 32 |
| CSF1R-autoinhib. | 39 | FLT3 (D835V) | **98.9** | RET (V804M) | 8 |
| CSK | 20 | FLT3 (D835Y) | 25 | ROS1 | 23 |
| CTK | 56 | FLT3 (ITD) | 0 | SRC | 8 |
| DDR1 | 3 | FLT3 (ITD, D835V) | 60 | SRMS | 48 |
| DDR2 | 54 | FLT3 (ITD, F691L) | **65** | SYK | 27 |
| EGFR | 15 | FLT3 (K663Q) | 0 | TEC | 3 |
| EGFR (E746-A750 del) | 21 | FLT3 (N841I) | 6 | TIE1 | 0 |
| EGFR (G719C) | 10 | FLT3 (R834Q) | **69** | TIE2 | 39 |
| EGFR (G719S) | 24 | FLT3-autoinhib. | 43 | TNK1 | 61 |
| EGFR (L747-E749 del, A750P) | 10 | FLT4 | 7 | TNK2 | 9 |
| EGFR (L747-S752 del, P753S) | 26 | FRK | 18 | TRKA | 22 |
| EGFR (L747-T751 del, Sins) | 0 | FYN | 4 | TRKB | 55 |
| EGFR (L858R) | 4 | HCK | 16 | TRKC | 30 |
| EGFR (L858R, T790M) | 49 | IGF1R | 2 | TXK | 22 |

**Supplementary Table S2 (continued)**

| Kinase Abbreviation | % | Kinase Abbreviation | % | Kinase Abbreviation | % |
|---|---|---|---|---|---|
| EGFR (L861Q) | 0 | INSR | 53 | TYK2 (JH1 dom. cat.) | **74** |
| EGFR (S752-I759 del) | 24 | INSRR | 0 | TYK2 (JH2 dom. pseudokin.) | 64 |
| EGFR (T790M) | 63 | ITK | 17 | TYRO3 | 13 |
| EPHA1 | 21 | JAK1 (JH1 dom. cat.) | 3 | VEGFR2 | 63 |
| EPHA2 | 16 | JAK1 (JH2 dom. pseudokin.) | 11 | YES | 5 |
| EPHA3 | 0 | JAK2 (JH1 dom. cat.) | 39 | ZAP70 | **76** |

Abbreviations: P, phosphorylated form; non-P, non-phosphorylated form; cat., catalytic; term., terminal; kin., kinase; dom., domain. Residues are indicated in brackets for mutated forms. The percentage of binding inhibition for the 10 kinases for which ≥65% inhibition of ligand binding was observed is shown in bold.

**Supplementary Table S3.** Partial docking scores of ten highly interacting residues for the docking poses of compounds **3h** and **3g** in 6JQR (D835V) structure (DFG-in conformation).

| Compound **3h** | | Compound **3g** | |
|---|---|---|---|
| **Residue** | **$DS_p$ [a]** | **Residue** | **$DS_p$ [a]** |
| Val624 | -18.511 | Val624 | -20.122 |
| Leu818 | -15.792 | Leu616 | -17.234 |
| Leu616 | -15.315 | Leu818 | -15.543 |
| Cys828 | -12.866 | Cys828 | -13.865 |
| Cys694 | -10.966 | Lys644 | -11.145 |
| Lys644 | -10.430 | Phe691 | -9.252 |
| Asp829 | -9.638 | Ser618 | -8.854 |
| Phe691 | -9.165 | Gly617 | -8.306 |
| Ser618 | -7.789 | Asp829 | -6.695 |
| Gly617 | -7.611 | Gly697 | -6.116 |

[a] A unitless value of partial docking score calculated with MolDock force field.

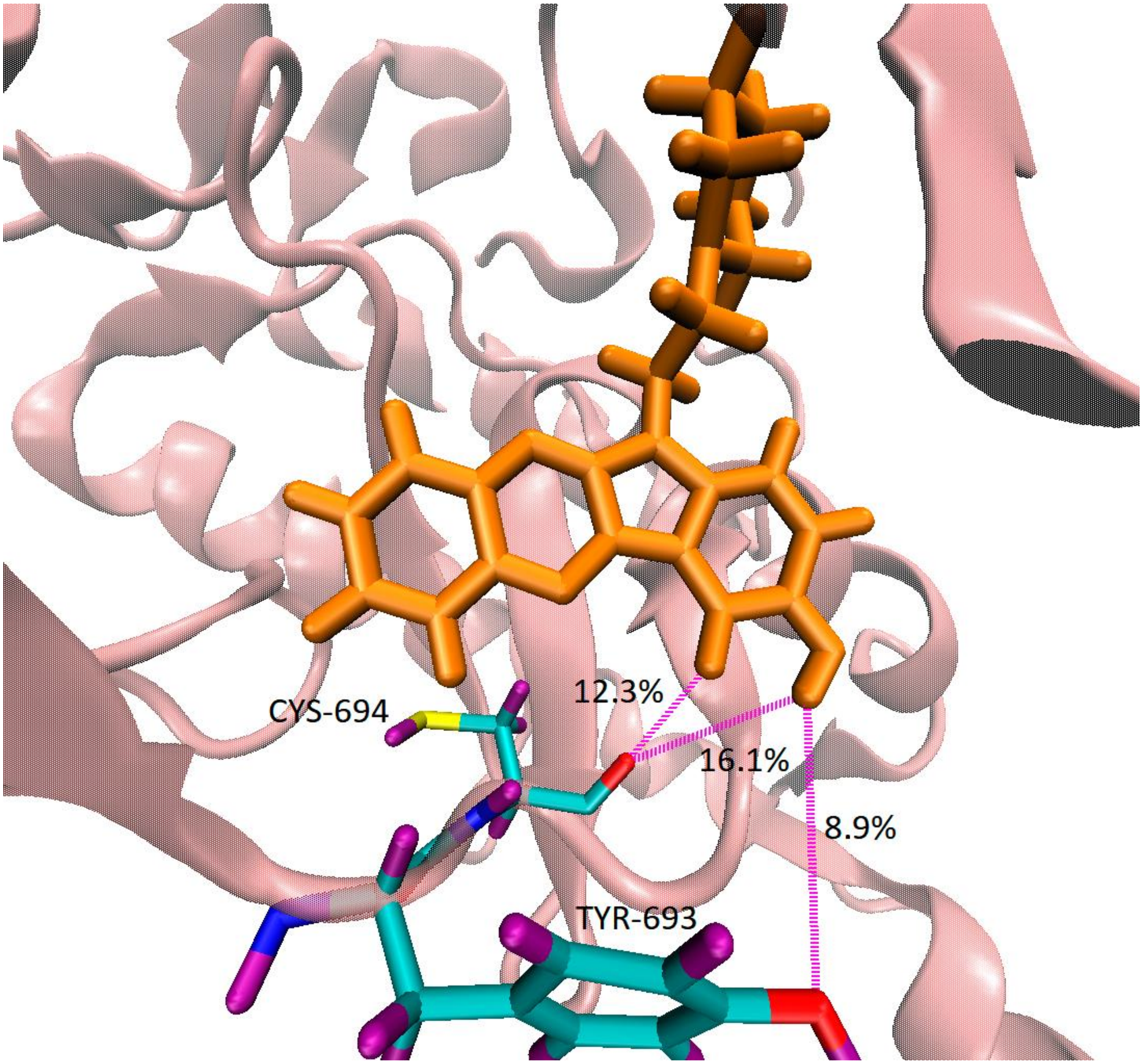


**Supplementary Figure S1**. MD-predicted orientation of compound **3h** in the ATP-binding site of D835V mutant FLT3. Purple dashed lines represent the most persistent hydrogen bonds.
